# Co-designing solutions to enhance access and engagement in pediatric telerehabilitation

**DOI:** 10.3389/fresc.2023.1293833

**Published:** 2023-12-20

**Authors:** Meaghan Reitzel, Lori Letts, Cynthia Lennon, Jennifer Lasenby-Lessard, Monika Novak-Pavlic, Briano Di Rezze, Michelle Phoenix

**Affiliations:** ^1^ School of Rehabilitation Science, Faculty of Health Sciences, McMaster University, Hamilton, ON, Canada; ^2^ CanChild Centre for Childhood Disability Research, McMaster University, Hamilton, ON, Canada; ^3^ KidsAbility Centre for Child Development, Waterloo, ON, Canada; ^4^ Parent Partner, Waterloo, ON, Canada; ^5^ Psychology Department, University of Guelph, Guelph, ON, Canada; ^6^ Bloorview Research Institute, Holland Bloorview Kids Rehabilitation Hospital, Toronto, ON, Canada

**Keywords:** childhood disability, experienced based co-design, health service research, pediatric telerehabilitation, service access, service engagement

## Abstract

**Introduction:**

Prior to the COVID-19 pandemic, children's therapy appointments provided by Ontario's publicly-funded Children's Treatment Centre (CTCs) primarily occurred in-person. With COVID-19 restrictions, CTCs offered services via telerehabilitation (e.g., video, phone), which remains a part of service delivery. CTC data shows that families experience barriers in attending telerehabilitation appointments and may need supports in place to ensure service accessibility. Our study aimed to co-design innovative solutions to enhance access and engagement in ambulatory pediatric telerehabilitation services. This manuscript reports the co-design process and findings related to solution development.

**Methods:**

This research project used an experience based co-design (EBCD) approach, where caregivers, clinicians and CTC management worked together to improve experience with telerehabilitation services. Interview data were collected from 27 caregivers and 27 clinicians to gain an in-depth understanding of their barriers and successes with telerehabilitation. Next, 4 interactive co-design meetings were held with caregivers, clinicians and CTC management to address priorities identified during the interviews. Using qualitative content analysis, data from the interviews and co-design meetings were analyzed and findings related to the solutions developed are presented.

**Findings:**

Four topics were identified from the interview data that were selected as focii for the co-design meetings. Findings from the co-design meetings emphasized the importance of communication, consistency and connection (the 3C's) in experiences with telerehabilitation. The 3C's are represented in the co-designed solutions aimed at changing organizational processes and generating tools and resources for telerehabilitation services.

**Discussion:**

The 3C's influence experiences with telerehabilitation services. By enhancing the experience with telerehabilitation, families will encounter fewer barriers to accessing and engaging in this service delivery model.

## 1. Introduction

In 2006, 174,810 Canadian children aged 5–14 years had a disability as per the Participation and Activity Limitations survey criteria (1). According to a report released by Statistics Canada in 2022, 13.5% of Canadian children aged 0–14 were reported to experience at least one activity limitation as a result of a difficulty or long-term condition (e.g., mobility, learning, emotional/psychological) (2). Rehabilitation services help children with disabilities achieve functional outcomes and participate in their social environments (3–5). Annually, publicly-funded Children's Treatment Centres (CTCs) in the Canadian province of Ontario provide over 750,000 rehabilitation visits to children (ages 0 to age of secondary school exit) and their families (6). These rehabilitation services include a combination of occupational therapy (OT), physical therapy (PT), speech-language pathology (SLP) and social work (SW) services. Some CTCs also employ Board Certified Behaviour Analysts (BCBA) and Instructor Therapists (IT) to provide services to autistic children. The term clinician is used throughout this paper and could refer to a care provider from any of the previously mentioned disciplines.

CTCs provide ambulatory services based in treatment centres to address home and community goals; however some also provide services in the school setting. Prior to the COVID-19 pandemic, CTC appointments primarily occurred in-person. COVID-19 restrictions limited access to in-person rehabilitation services and children's rehabilitation service providers quickly pivoted to supporting families using telerehabilitation platforms (7). Prior to the COVID-19 pandemic it is estimated that only 4% of pediatric care clinicians used telerehabilitation; this number drastically increased to 75% during the pandemic (7). Given the rapid uptake of telerehabilitation during the pandemic, there have been calls to consider its potential to be integrated into a hybrid service model, that takes into account reported benefits of offering a combination of in-person and telerehabilitation services (7, 8).

Telerehabilitation is defined as therapy occurring remotely over a telecommunication platform such as telephone or video conferencing (9). Increasingly, telerehabilitation services are being provided by allied health clinicians and are proposed as a solution to barriers encountered when accessing in-person rehabilitation services, such as the time and cost associated with travelling to appointments (7, 10). In a 2023 systematic review examining the effectiveness of telerehabilitation in children with developmental disabilities, telerehabilitation was found to be more effective when compared to no treatment for outcomes such as functional performance, hand function, visual perception, and behaviour or as effective when compared to no treatment (i.e., waitlist) and usual treatment, respectively (11). For outcomes such as, self-efficacy, self-control and social skills, telerehabilitation was found to be as effective when compared to usual treatment (11). In autistic children, telerehabilitation was found to be more effective than in-person services across 85% of outcomes and most importantly, telerehabilitation was never found to be less effective or to cause harm (11). This evidence of effectiveness aligns with findings from another systematic review that described telerehabilitation as an effective approach to supporting the development of adaptive skills in children with multiple disabilities (12). The benefits and challenges of telerehabilitation in outpatient pediatric rehabilitation services during the COVID-19 pandemic have been described and it is recommended that service organizations address barriers to optimize the effectiveness of this model of care (13).

Evidence demonstrates that therapy outcomes and experiences are enhanced when families are actively engaged with the services they receive (14–16). Family engagement in therapy is supported through a shared understanding of expectations, collaboration and positive relationships with therapists (15, 17). To date much of the telerehabilitation literature examines its effectiveness (11, 18, 19) and the acceptability of this service model from the caregiver perspective (7, 20), however qualitative research has started to explore parent engagement in telerehabilitation as it relates to the parent-therapist relationship (10). In 2022, a qualitative systematic review described engagement in early intervention telerehabilitation for young children with developmental disabilities and provided recommendations to establish and maintain engagement with these services (21). Despite this emerging evidence related to engagement in telerehabilitation, little is known about whether telerehabilitation can assist families in attending appointments and improve engagement in their child's therapy. The Phoenix Theory of Attendance, Participation and Engagement (the Phoenix Theory) has provided substantive knowledge regarding the barriers families experience accessing, participating and engaging in CTC services when offered in-person (15). This theoretical framework and associated research findings have been used successfully at our partner CTC (KidsAbility) to inform organizational policies and services affecting families who miss in-person appointments to reduce barriers to service access and engagement.

Missed appointments are defined as appointments missed without prior notification to the CTC and have been problematized as inefficiently using clinician time and organizational resources and may impact therapeutic outcomes (22, 23). For this project, we partnered with KidsAbility Centre for Child Development (KidsAbility), an Ontario CTC to explore missed telerehabilitation appointments, defined by KidsAbility as appointments occurring by phone or video. Since commencing with telerehabilitation services in March 2020, KidsAbility continues to report high numbers of missed appointments. From 2022 to 2023 14% (*n* = 1,652) of telerehabilitation appointments were missed, which was comparable to 15% (10, 349) of in-person appointments that were missed at KidsAbility. A total of 456 telerehabilitation appointments were missed without prior notice, limiting opportunities for clinicians to effectively use that client time. These metrics indicate that families experience barriers to service use, even when services are offered via telerehabilitation.

The aim of this project was to co-design innovative solutions that will enhance access and engagement in telerehabilitation in the context of publicly-funded pediatric rehabilitation for children with disabilities. We have collaborated with KidsAbility and a parent-partner to address the following research question: What co-designed solutions can be developed to improve families' access and engagement in pediatric telerehabilitation services? The scope of this paper focuses on describing the co-design process and reports findings related to the solutions developed.

## 2. Materials and methods

### 2.1. Study design

Experience based co-design (EBCD) is a highly collaborative approach to research that focuses on the lived experience of service users and service providers to develop innovative solutions to health service issues (24, 25). EBCD necessitates authentic engagement with invested parties (caregivers, clinicians, health service organizations) throughout research development, implementation and evaluation (25, 26). With its origins in design sciences, EBCD has been proposed as an approach to create or modify health service experiences through integrating patients as partners in service design projects (27). EBCD has been utilized to design health services in the public sector with the potential for authentically engaging vulnerable populations (24, 28–30).

EBCD prioritizes collaboration, partnership between invested parties and researchers, lived experience as expert knowledge, capacity building and creativity in generating solutions (25, 27, 29). Integrating qualitative methods, this project is guided by the six stages of the EBCD approach proposed by Bate and Robert (2007). For the purpose of this project, the stages of EBCD were conceptualized as: (1) setting up the project; (2) engaging clinicians and gathering their experiences; (3) engaging families and gathering their experiences; (4) co-design meetings; (5) sustain co-design engagement and implement change; (6) celebrate and evaluate changes to health service.

Stages 1 through 4 all contribute to the overall co-design process. This paper will provide a detailed account of the methods and findings for the stage 4 co-design meetings, when the co-designed solutions were developed. Stages 1 through 3 will be reviewed briefly with a focus on how they informed the stage 4 co-design meetings. Table 1 provides a summary of key information linked to stages 1 through 4 of the co-design process as related to this project. Ethical approval for this study was received by the Hamilton Integrated Research and Ethics Board (project #14235).

**Table 1:** Summarizing stages 1 through 4 of co-design process.

	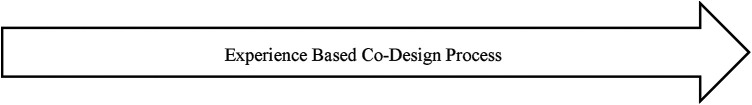			
	Stage 1—setting up the project	Stage 2—engaging caregivers and gathering their experiences	Stage 3—engaging clinicians and gathering their experiences	Stage 4—co-design meetings
Purpose	Establish channels to advise project directions from the perspective of multiple invested parties	Understand experiences with receiving telerehabilitation at KidsAbility	Understand experiences with providing telerehabilitation at KidsAbility	Co-design solutions to enhance telerehabilitation experience
Participants	Steering committee: parent (*n* = 1), clinicians (*n* = 2) researchers (*n* = 4), KidsAbility Parent Advisory Committee: (PAC) (*n* = 6 members consulted)	Caregivers (*n* = 27)	Clinicians (*n* = 27)	Caregivers (*n* = 9), clinicians (*n* = 12), managers (*n* = 3) Groups ranged from 5 to 7 participants
Data collection	Parent Advisory Committee: Single point of consultation during project conceptualization Steering committee: Consultation throughout the project to develop the research question, methods, and participate in data collection and analysis	Interviews	Interviews	In-person co-design meetings (*n* = 3) virtual codesign meeting (*n* = 1)
Outcome(s)	Research methods and findings tailored to KidsAbility practice context	Touch point identification to inform co-design meetings	Touch point identification to inform co-design meetings	3Cs (communication, consistency, connection) impacting telerehabilitation experience and co-designed solutions to improve access and engagement in telerehabilitation

### 2.2. Study context

The study context is described in detail to aid readers in determining the transferability of our findings to other settings. This study was completed at KidsAbility in Ontario, Canada. KidsAbility has 6 sites (5 permanent locations and 1 rural satellite clinic) providing publicly-funded children's rehabilitation services across a highly multicultural region that includes both urban and rural communities. In response to restrictions associated with the COVID-19 pandemic, KidsAbility pivoted to providing telerehabilitation services, which continue to be offered as part of a hybrid service model combining both in-person and virtual visit options. A partnership was formed with KidsAbility for this project because author MR worked there as a clinician, facilitating a deep understanding of the culture, services, provision of telerehabilitation and characteristics of the families served. Author MR examined the impact of her dual role as a clinician and a researcher who is closely connected to the study context by engaging reflexively with literature on this topic, keeping reflective memos and by debriefing with the steering committee, to ensure multiple perspectives were included in all project decisions.

### 2.3. Stage 1: setting up the project

The need to reduce barriers in accessing telerehabilitation services was identified from the results of a survey administered by KidsAbility in 2020. Survey results aligned with concerns that were raised by the KidsAbility's parent advisory committee related to families' equitable access and engagement in telerehabilitation services. The parent advisory committee is a voluntary committee of caregivers whose children are currently engaged with services at KidsAbility or had received services in the past. Open discussion forums were held with the parent advisory committee to guide the development of the research question, objectives, and to identify meaningful indicators of access and engagement in telerehabilitation services. Insights from six committee members emphasized the importance of diverse family representation in the study including geography, ethnicity, family composition, and characteristics of the child (e.g., severity of needs and age) presuming that barriers to telerehabilitation would vary according to these factors.

A multi-disciplinary steering committee including four interdisciplinary researchers, two individuals with clinical experience providing telerehabilitation services and a parent whose child had received KidsAbility services was assembled. The steering committee meets regularly and is responsible for collaboratively participating in all aspects of the project, including but not limited to defining the research question, establishing methods for data collection, engaging in data collection, facilitating co-design groups, supporting data analysis and contributing to knowledge sharing activities (e.g., presentations, manuscript preparation).

### 2.4. Stages 2 and 3: engaging clinicians/caregivers and gathering their experiences

The data from semi-structured interviews completed with 27 caregivers and 27 clinicians about their experiences with telerehabilitation will be reported in a separate paper. These interviews informed the co-design process by eliciting the touch points, which are emotionally powerful and memorable highs and lows of engaging in telerehabilitation (27). Interviews were completed virtually and audio recorded using the Zoom platform (31) between October 2022 and December 2022. Inductive qualitative content analysis was completed to identify, describe and visualize the touch points. Following this analysis, MR led the steering committee in a journey mapping elicitation activity where Google Jamboard was used (32) to further categorize touch points based on commonalities and to map them onto a timeline representing the journey of a telerehabilitation appointment (i.e., time leading up to the appointment, during the appointment and follow up from the appointment). The purpose of this task was two-fold. First, mapping the touch points provided a visual depiction of when participants were experiencing the touch points during their telerehabilitation journey. Second, through collaborative discussion, journey mapping allowed for the prioritization of the touch points that would be carried forward into the co-design meetings aimed at developing solutions to enhance the telerehabilitation experience. An audit trail was kept to document decisions made by the steering committee during all data collection and analysis phases of this project. Analytic memos documenting reasoning for decisions and directions taken during this project were kept by author MR. Peer debriefing was practiced during monthly meetings with the steering committee to guide project related decisions.

### 2.5. Stage 4—co-design meetings

#### 2.5.1. Sampling

Caregivers with children who received telerehabilitation services from KidsAbility in the previous 12 months were recruited by self-referral using established communication channels between KidsAbility and families (e.g., KidsAbility's social media platforms, website and email list). Direct emails to clinical staff and advertising in the internal staff newsletter were used as additional strategies to recruit clinicians via self-referral who had provided telerehabilitation service KidsAbility in the previous 12 months. The timeframe of 12 months was selected for both caregivers and clinicians to ensure that they had relatively recent experiences receiving or providing telerehabilitation. The desire was for experiences to be representative of the current status of telerehabilitation service provision and not of that which was provided when CTCs were required to pivot to this unfamiliar service model in response to the COVID-19 pandemic in March 2020.

Participants were recruited to take part in one of the four co-design meetings. Given that our aim was to maximize the diversity of perspectives, caregiver and clinician participants did not have to complete an interview in stages 2 or 3 to participate in the stage 4 co-design meetings. In addition to clinicians and caregivers, managers who directly supervised staff providing telerehabilitation services were also recruited for this stage of the co-design process. Managers were recruited through the same internal communication channels as clinical staff (i.e., internal newsletter, email). Although managers did not have direct experience providing telerehabilitation services at KidsAbility, co-design approaches recommend including those in positions to influence service delivery decisions (25). Therefore, our steering committee felt it was important that managers be included in the development of solutions to the touch points identified in stages 2 and 3. Including managers ensured their voice was heard in the process and encouraged investment in the co-designed solutions, enhancing implementation and sustainability efforts. Recruitment for this phase of the project launched in February 2023 and closed April 2023.

#### 2.5.2. Participants

Sixteen caregivers were enrolled into this phase of the study and 9 attended a co-design meeting as planned (one parent could not be reached to schedule into a meeting, one parent cancelled prior to the scheduled meeting and 5 did not give prior notice that they would not be attending). Demographic data were collected using a form developed in Research Electronic Data Capture (REDCap) (33). Caregivers were recruited from 3 of 6 KidsAbility sites, with one family reporting that they lived rurally. Seven families identified that the primary language spoken in the home was English, while the two other families spoke either Telugu or Bilen. All families identified having access to reliable internet at home. Seven mothers and 2 fathers participated in the co-design meetings and all families identified having one child who received telerehabilitation services from KidsAbility. Children of the caregiver participants ranged in age, 0–3 years old (*n* = 4), 4–7 years old (*n* = 4) and 12–15 years old (*n* = 1). Caregivers identified their children as having the following diagnoses: speech and language delay (*n* = 5), global developmental delay (*n* = 3), autism spectrum disorder (*n* = 2), cerebral palsy (*n* = 1), and other (sensory processing differences, epilepsy) (*n* = 2). Two families reported that their child had more than one diagnosis. Six families engaged in telerehabilitation appointments with SLP, 5 with OT, 2 with PT, 2 with SW and 1 family was unsure of the clinical discipline they interacted with. Four families received telerehabilitation from more than one clinical discipline and all families reported that these were individual sessions with their child. One family indicated receiving both group and individual therapy.

Thirteen clinicians enrolled and 12 participated in a co-design meeting (one clinician was unable to attend due to a change in their availability). Representation of clinical disciplines included SLP (*n* = 7), CDA (*n* = 2), IT (*n* = 1), OT (*n* = 1) and PT (*n* = 1). Years of clinical experience of the clinical participants ranged from 1 to 5 (*n* = 5), 6 to 10 years (*n* = 4) and 11 to 15 years (*n* = 3). Six clinicians identified having 0 to 2 years of experience providing telerehabilitation services and 6 identified having 3 to 5 years of experience. Three managers were enrolled and participated in a co-design meeting. The participating managers reported having at least 16 years of clinical experience in their discipline, while management experience ranged from 1 to 5 years (*n* = 1), 6 to 10 years (*n* = 1) and 11 to 16 years (*n* = 1). Between clinicians and managers, participants represented all clinical programs at KidsAbility (e.g., early intervention services, school aged and school-based rehabilitation services, autism services, and specialized services such as augmentative communication services).

#### 2.5.3. Data collection and analysis

Four co-design meetings, each two hours in length, were conducted between April 2023 and May 2023. Three of these meetings were conducted in-person, at three different KidsAbility sites and one was held virtually over Zoom (31) to accommodate those who were unable to attend in-person. Three of the four co-design meetings had caregiver, clinician and management representation. One in-person group did not have a manager participate. All sessions were audio and video recorded to facilitate subsequent transcription and analysis of the data. Authors MR and MNP co-facilitated all meetings alongside a parent facilitator. All parent facilitators had experience being members of a research team and/or facilitating group discussions with other caregivers. The parent co-facilitator worked closely with the caregiver participants to validate their experiences, encourage idea sharing and create a safe space for collaboration. Transportation and language interpretation services were made available in all phases of this project to enhance the accessibility of participation.

The co-design meetings were run in an interactive focus group format. Each co-design meeting focused on a different touch point that emerged from interviews. The aim of the co-design meetings was to bring multiple invested parties (caregivers, clinicians and management) together to collaboratively develop solutions and prototypes for the touch points impacting experiences with telerehabilitation at KidsAbility. Each co-design meeting was divided into three sections: (1) introductions, orientation to the touch point and aims for the session; (2) solution development; and (3) prototype development. The COMPASS for Relational Safety in Co-design/Production and the corresponding MAPS framework guided the structure of the group to work toward creating an atmosphere where all participants felt comfortable collaborating toward a common goal (34).

(1) Introductions, orientation to the touch point and aims for the session—The meeting began with introductions and an ice breaker activity in the hopes of creating relatable moments between participants (34). Guidelines for engagement were discussed to ensure all participants had a common understanding of suitable ways to engage in discussion and idea sharing. Participants were oriented to the touch point of focus for their meeting using multimedia tools. These tools included an animated video depicting the positives aspects of telerehabilitation services as reported by caregivers and clinician during the interviews as well as a poignant image with a voice over of a caregiver and clinician speaking about the negative aspects of telerehabilitation in relation to the touch point. Once familiar with the touch point, the aims of the session and the activities were reviewed with the participants.
(2) Solution development—Next the participants were presented with the task of developing solutions to the touch point. A modified 1-2-4-all Liberating Structure was used to guide this activity whereby participants started with independent idea generation, shared ideas in small groups and then engaged in a full group discussion about the favourite ideas generated by each small group. Liberating Structures are a set of interactive methods used to facilitate inclusive engagement of multiple and diverse voices working toward a collective purpose and have been used to support change in health services research (35–37). Specifically, the 1-2-4-all Liberating Structure is an effective way to engage multiple people at the same time to generate ideas (36). Every participant was given a sticker to place beside their favourite idea and the idea with the most stickers was brought forward for further discussion in the prototyping phase.
(3) Prototype development—The idea that was prioritized for prototyping was the focus of section three of the meeting. Participants broke into their small groups and used arts-based methods (e.g., paper, sticky notes, markers, coloured stickers, etc.) to design low fidelity prototypes of what it would look like to implement the prioritized solution into the policy and practices of KidsAbility. Tools available in Jambord (32) (e.g., white board, sticky notes, labels) were used to support prototyping during the virtual meeting. Low fidelity prototyping is a technique described in the EBCD process (27). The participants then reconvened as a full group to provide verbal descriptions of their prototypes.

The aim of data analysis during stage 4 of the co-design process was to describe the solutions prioritized and the prototypes developed by participants in the co-design meetings. Data from the co-design meetings were analyzed using inductive qualitative content analysis as described by Elo & Kyngas (38). Data sources from the co-design meetings included sticky notes from the idea generation phase, the prototype materials (e.g., sketches) and transcripts from group discussions. Transcripts were read multiple times by author MR to make sense of the data. During a collaborative analysis session, authors MR and MP engaged in open coding and categorization of data from the transcripts, sticky notes and prototypes. Additionally, transcripts were coded and categorized by author MR using NVivo software (39) through line by line reading of the transcripts. Data from the transcripts contextualized the arts-based data (sticky notes and prototypes) by integrating explanations of the participants who generated the ideas. Data across all four focus groups were analyzed to explore similarities and differences in the solutions developed as well as potential opportunities to blend similar prototypes. Categorized was synthesized into narrative form by authors MR and MP via the use of analytic memos. Iterations of the narrative synthesis were reviewed during peer debriefing meetings between author MR and senior researcher MP. Member checking with the participants in the co-design meetings was not completed, however the categories and synthesis were reviewed and validated by authors JLL and CL through the caregiver and clinician lens respectively and feedback was incorporated into the findings. Their feedback did not result in altering the coding or categorization structure.

## 3. Results

The results of this research are described in four sections below. First, touch points identified from the interviews completed with caregivers and clinicians in stages 2 and 3 are summarized. A full account of the interview findings falls outside of the scope of this paper and will be reported in a future manuscript. Next, the findings from the analysis of the data collected from the stage 4 co-design meetings are described as the 3C's (communication, consistency, connection) in telerehabilitation experience. The co-design solutions developed to address the 3C's prior, during and after therapy are presented.

### 3.1. Touch point identification through sharing stories of telerehabilitation experiences

Four touch points were inductively identified from the caregiver and clinician experiences with telerehabilitation that were shared during the interviews. The four touch points identified were: (1) child engagement in telerehabilitation; (2) perceived value of telerehabilitation services and caregiver engagement; (3) fit of using a telerehabilitation model and providing family with choice; (4) preparing the people and environment for telerehabilitation services. Each touch point served as a topic for the four co-design meetings.

### 3.2. The 3C's in telerehabilitation experience—communication, consistency, connection

Open coding of the transcripts and analysis of the arts-based outputs (e.g., drawings, chart paper, sticky notes) from the four co-design meetings led to the identification of three interconnected categories identified as impacting the telerehabilitation experience. These three categories are communication, consistency and connection (the 3C's). All invested parties (i.e., caregivers, clinicians, management) involved in the co-design meetings identified examples of how challenges with the 3C's impact experiences with telerehabilitation at KidsAbility. A desire to improve how the 3C's are experienced by caregivers and clinicians is apparent in the co-designed solutions and related prototypes. Table 2 summarizes key information from analysis that describes the subcategories and categories related to the 3C's.

**Table 2:** Key components of the 3C's impacting experiences with telerehabilitation services.

	Categories		
	Communication	Consistency	Connection
Subcategories	About the telerehabilitation service model	In sessions between clinicians (e.g., format, quality)	Between treating clinician and family
	About the aims of the telerehabilitation session	In providing choice and flexibility in service	Should be established early on in service
	Should be multimodal and tailored to the family		Impacts buy-in and engagement in telerehabilitation services

#### 3.2.1. Communication

Caregivers, clinicians and managers recognized significant deficits in how the details of telerehabilitation as a service model were communicated. General information such as what is a telerehabilitation appointment (i.e., over video or phone), what occurs during a telerehabilitation appointment and what technology/set up is required for a telerehabilitation appointment was not adequately reviewed with caregivers prior to commencing with service. “Communication is the biggest key in all of this, it's lacking at some point or points. A new person coming in, jumping right to virtual…with no further communication, they're going to be lost.” (Caregiver P1-2). A caregiver recalling her initial telerehabilitation appointment shared, “I remember my first session, and it was just chaos… (Caregiver P2-2). Without adequate communication prior to initial and subsequent telerehabilitation appointments, caregivers expressed feeling unprepared for the sessions, which impacted how meaningful the session was perceived to be, “If there was some sort of communication prior: this is what speech needs to see, this is what OT needs to see, let's do this activity because we can see both…. There was none of that, and it was overwhelming, and at the end of it, I was like, “Okay, cool, what did we accomplish?” (Caregiver P1-2). “If they had sent an email ahead of time that said, ‘Hey, you can have snacks or something ready?’ Then yep, I could have had it in place” (Caregiver P2-2).

Clinicians also identified the importance of communicating the aims of the session so that families could join feeling prepared, “having the family aware, if I want to see your kid in a walker, it can’t be in storage, you have to have it ready for the session. So, preparing everyone beforehand, and then giving them the tools based on what we’re hearing” (Clinician P7-4). Specific mention was made about the importance of ensuring clear and accessible communication about telerehabilitation services for families when English is not the primary language spoken. The need for “supporting parents for whom English is a second language…all the way through” (Parent Facilitator P3-3) including support for communicating with KidsAbility, accessing technology for telerehabilitation and teaching strategies for supporting caregivers to engage children in telerehabilitation appointments.

Communication impacted caregivers' expectations of therapy services. Caregivers identified feeling that there was a lack of communication provided to help inform them of what to expect with regards to wait times for visits and how many visits they could expect to receive, “I was on a waitlist for about a year, and I got one online session for an hour and that was it. I thought this was a long wait for nothing…My expectations were up to here. I got shafted.” (Caregiver P3-4). A lack of clarity was also identified regarding the caregiver's role during a virtual appointment. Sharing one of her experiences with a telerehabilitation appointment, a caregiver stated, “I remember I did one therapy session, and they needed me to actually measure his spasticity. I was not prepared for this,…nobody told me that's what I’d be doing this virtual session.” (Caregiver P4-1). All stakeholders identified the need for communication between the clinician and caregiver prior to commencing with a telerehabilitation session to help ensure all involved felt prepared and shared the same expectations for the appointment. “There's pre-work for the child and pre-work for the household and pre-work for the clinician. Are the 2 entities aligned in what's to be expected?” (Caregiver P4-1). The importance of matching therapy expectations is highlighted in these statements from clinician and manager participants, “Before you start a therapy, we [participant group] thought not only that the parents recognize the expectation that if this is a virtual service, you’re going to need to do XYZ, but also, in return, that we’re understanding what they’re expecting from the service.” (Clinician P1-3). “If everyone has the same expectation and is able to have done the work beforehand for that session, then you're going to be able to have a lot more success with the session rather than one person be disappointed.” (Manager P2-1).

The mode of communication was also highlighted by caregivers as critical to consider when establishing effective communication between KidsAbility and families. When discussing modes of communicating one parent expressed, “My biggest point that I keep saying here is that emails get lost…Trying to go back for something that took place 3 months ago in emails, like where is that document? I know it's here somewhere. It's hard, right? So I wouldn't suggest an email touching base by any means. I think a phone call would be more efficient, ahead of time, before you got on to the link [for the telerehabilitation appointment].” (Caregiver P1-2). A clinician participant shared the following reflection about their experience sending emails to caregivers prior to telerehabilitation appointments, “…less and less parents are prepared because I think what's happening is there's just too much information. So, I think having that discussion versus an email would be helpful to really make sure we're on the same page about what this is going to look like.” (Clinician P3-2). The importance of a “multimodal approach to communication” (Clinician P7-4), was recognized with an understanding that “some people may want to phone call, some people want to email,…asking how they best communicate…Adding a multimodal approach is what you'd need, considering how we can best deliver the information” (Clinician P7-4).

In addition to establishing a preferred mode of communication, tailoring the amount of information shared was also discussed as an important aspect of communication impacting experiences with telerehabilitation. A lack of communication prior to a telerehabilitation visit left caregivers feeling unprepared, while high volumes of information shared in follow up to an appointment was expressed to feel overwhelming. One mother shared this narrative about information that was provided after a telerehabilitation session: “My baby is medically fragile—that's one set of needs. And my eldest is on the spectrum [autism]. After one particular session, I was just inundated with information, and it was so overwhelming at the time because I had a baby and then a 2-year-old…But I was told, go watch this video, go on to this link, and then there were multiple attachments of 50-page documents of resources. I was so overwhelmed, but so desperate to have my husband and I help our 2-year-old” (Caregiver P5-4). Another caregiver said “I did get an email after my one call, with a whole bunch of resources…I thought this may be relevant and that, but it was so big that I just thought I would get back to that eventually, and I never did because it was overkill” (Caregiver P3-4).

#### 3.2.2. Consistency

The importance of consistent practices and processes related to telerehabilitation services across clinicians and KidsAbility programs was identified by co-design meeting participants as another area instrumental in influencing experiences engaging with these services. Some caregivers had experience engaging in telerehabilitation services with multiple clinicians and reported that practices across clinicians varied. “So, I've done Zoom with 4 [different clinicians], and they are all completely different, and there is no consistency whatsoever in the way that they do it.” (Caregiver P1-2). During a co-design meeting, a clinician shared the approach they took to support families in preparing for a virtual session, which according to caregiver participants, varied greatly from what they experienced with the clinicians they worked with, “It's just crazy that other people did it so differently, and it was so much more beneficial” (Caregiver P1-2). “I'm just going to say, from a parent's perspective, if there was that kind of training, it might help us on the consistency that we thought we would get” (Caregiver P2-2). Clinicians acknowledged inconsistencies in practice, “I don't even know what happens in other virtual sessions. I know what happened in my virtual sessions, but you're right. If there was some consistency…it would be more clear for everyone.” (Clinician P4-2). Clinicians also recognized value in there being a “clear stepwise process, internally, for therapists, so that it's more consistent” (Clinician P4-2).

A desire for consistent choice and flexibility integrated into telerehabilitation service delivery was highlighted by caregivers and clinicians when discussing service experiences. A clinician described using a flexible approach to learn about how caregivers would choose to design telerehabilitation, “I had some success in the past with discussing with the parents and saying, ‘How do you like to learn? How do you want this session to go?’… Do you like to learn the strategy on your own in a discussion format just with me and then the next week, your child can attend?” (Clinician P3-2). In contrast to the flexibility described by the clinician, a caregiver attributed their negative experience to a lack in choice regarding how telerehabilitation visits were conducted, “So, I do joint speech and occupational therapy at the same time… And I’ve tried very hard to get out of having to do my sessions together, to do them separately, which I've not been successful with. They keep doing it.” (Caregiver P1-2). “There was also some discussion around when KidsAbility calls to make an appointment, whether the parent could decide at that time, ‘I'd like this appointment to be virtual, or I think I can make it in person,’ whether that level of flexibility could be provided, so that isn't a decision that we're making blanket from the beginning. But when the appointments are scheduled, we can sort of think through whether at that time it might be more appropriate to do a virtual or in person.” (Parent Facilitator P3-3).

#### 3.2.3. Connection

Developing a connection between the clinician and family early on in service engagement was identified by caregivers as being critical to their experience with telerehabilitation services. Caregivers described connection as feeling like their clinician knew about their child and family beyond the therapeutic context, that the clinician valued caregiver input and the clinician collaborated with the caregiver in a partnership. “There has to be some connection built with the families as a whole. The parents and the children. You can’t, for your first time, go on virtual, which we did, and expect the kids to listen and to cooperate and be comfortable to move forward” (Caregiver P1-2). Prior to commencing therapy involving the child, caregivers identified opportunities for building rapport with the clinician through early communication in the form of conversations about topics like what they are hoping from therapy, preferences for how visits occur and goal setting. “There still needs to be that connection with your therapist, more from the get-go” (Caregiver P1-2). When discussing goals, a caregiver shared, “So I think the goal setting is really important. The clinician obviously has that background, they are the professional, and they know what the goals are, but as a parent, that might not be the goal that you have for your child. It's probably still on there, but it might be number 10 on your list, but number one for your daily life and for the success of your child and your family unit might be a different goal that you're [the clinician] hoping to gain.” (Caregiver P5-4). The importance of following the family's lead in identifying priorities for therapy was also recognized by KidsAbility staff, “what do the parents want? What are you trying to get out of this? That's what we need to focus on” (Manager P2-4).

When caregivers feel that they are in a safe space with a strong connection to the therapist they were more confident in sharing information about their child (e.g., interests, likes, dislikes) and therapy preferences. It is important that clinicians invite this connection-building dialogue with caregivers as caregivers may fear repercussions for speaking negatively about their experiences with services. “I didn't want to rock the boat because I had waited for so long that I didn't want to lose that opportunity for her [child]” (Caregiver P2-2). A caregiver participant recognized that often the invitation to have these initial connection-building conversations are not consistently extended to families, “We don’t ask the parents what's overwhelming about this for you? It's all overwhelming, but what feels possible?… sometimes we don't check in on what do you [caregiver] need… Because if the parents are checked out,…you’re not getting the child” (Caregiver P4-1). By taking the time to connect with caregivers, clinicians can learn things about the child that may enhance engagement in therapy sessions. As an example a caregiver shared, “whenever my kid is excited, accomplished even a small task, sitting next to her, you just high-five. That may be something that parent and clinician can talk about…so that can keep them pumped and motivated to be engaged” (Caregiver P3-1).

The impact of connection on experience with telerehabilitation services was also recognized by clinician participants. “If we're asking questions, then hopefully, we're getting information. And then they're feeling that buy-in” (Clinician P8-4). “It sends the message that KidsAbility cares about your family, if they’re wanting to know things that aren't necessarily to do with their specific therapy. It's about you and your family and your child” (Clinician P1-4). Clinicians felt that service would be improved by “making that a standard, so that everyone just does these things to build rapport with your families, and really tailoring their service to that individual, feeling them out and building a relationship” (Clinician P1-4).

### 3.3. Co-designed solutions for improving the 3C's to enhance experiences with telerehabilitation

Solutions were co-designed by participants to address the 3C's (1) before; (2) during; and (3) after the visit. The solutions and related prototypes developed during the co-design meetings targeted these three parts of the journey, with a heavy emphasis on what can be done to support families and clinicians before the visit takes place. “That first pre-work will determine the format, the style, the extra things to get your child's attention. So for me, you’ve got to start at the beginning of the journey” (Caregiver P4-1).

The co-designed solutions are presented according to where participants felt they fit into the telerehabilitation journey. The solutions target either modifying the process related to engaging in telerehabilitation services at KidsAbility or developing a tool/resource that facilitates information sharing/gathering.

#### 3.3.1. Before the visit

Both process and tool/resource solutions were co-designed to promote consistent connection and communication between clinicians and caregivers when beginning telerehabilitation. To ensure there was a consistent opportunity for early communication and connection development, participants recommended implementing a process whereby clinicians book an initial appointment (likely by phone or video) with only the caregivers present. Caregivers expressed that this type of appointment would give them an opportunity to share information about their child as a person (e.g., likes, dislikes, motivators, interests, personality traits) and speak openly about their concerns and priorities for therapy. Clinicians saw additional value in the opportunity to connect with caregivers prior to commencing with telerehabilitation as it would give them a chance to have a conversation about the options for service models, learn about the caregiver's preference for services (examples identified by caregiver participants included: gender of clinician, ethnicity of clinician, appointment time/frequency/length), and make a service plan tailored to the family. In addition to occurring prior to commencing with therapy, participants recommended that this type of parent only appointment take place any time there is a change in treating clinician or when families are moving from in-person appointments to a telerehabilitation platform.

Participants prototyped tools/resources that included questions and discussion topics that clinicians could use during the pre-appointment conversation. Questions included: do you have access to the required technology and a reliable internet connection? Would you benefit from having an interpreter present? What are your goals for therapy? Here is what to do if we get disconnected from our visit. It was thought that a tool like this could act as a decision support when deciding what approach to take for therapy visits. Caregivers recommended consistent use of a “get to know my child” form to support the clinician in getting to know things like the child's likes/dislikes, which then can be integrated into therapy sessions to support engagement. “That [Get to Know my Child Form] would include things like your child's likes and dislikes, knowing what their dislikes are is equally as important as going through the long list of things they do like, their favorite toys, people in their life… So we're talking a lot about how to get your child engaged to be part of these [telerehabilitation session]” (Caregiver P4-1). Low fidelity prototypes of an online portal where parents and clinicians could directly message, share resources and update documents such as the “get to know my child” form was discussed as a possible platform to enhance communication between clinicians and families.

As another solution for enhancing early communication between the organization and families, participants prototyped the idea of video and text-based resources to share information with families about what they can expect when engaging in telerehabilitation appointments. Participants envisioned these resources being provided to families to support them in making informed decisions about what service model (i.e., in-person, virtual, combination) would feel like a fit for them. Videos would include footage of what a telerehabilitation session looks like, discuss technology requirements and environmental set up as well as review the caregiver's role during these sessions. “Video tutorials meaning tutorials explaining for families what a virtual appointment could look like based on the child's age, their situation, their environment, their goals… We thought this was important because we’re looking at some families thinking “virtual” means my child has to sit at the computer and engage in a computer game, and that's not always what we mean when we say virtual services for a child” (Clinician P2-3). Recommendations were made that these resources should be easily translated into a variety of languages to enhance accessibility.

#### 3.3.2. During the visit

The primary codesigned solution for during the visit targeted the consistency in communication through a process where clinicians summarize key points from the session and develop a plan for the next session that aligns with families' priorities. The aim of this solution is to establish a process to ensure that families complete the session with strategies they felt comfortable trying at home and an understanding of what they needed to have set up to feel prepared for the next session. “It's the prep for the next visit if that makes sense. It's developing that action plan and that take-home” (Clinician P1-1). This process creates consistent opportunities for clear communication and shared expectations about upcoming appointments.

#### 3.3.3. After the visit

Participants co-designed a process for follow-up after an appointment or block of sessions that facilitated authentic and individualized information sharing and communication methods. This solution was driven by caregivers' experiences of receiving emails in follow up to a visit with large amounts of content containing strategies and resources that felt generic. Participants recommended that in conversation with caregivers, clinicians inquire about preferred formats of receiving communication as well as the amount of information a caregiver prefers to receive. Caregivers made recommendations for “a more streamlined approach to the follow up. If it is resources and videos, ensuring that the parent has time to be able to view those and read over it. Having different ways of presenting material that isn't an email…” (Caregiver P5-4). A process to streamline how families engaged in telerehabilitation can access physical resources (e.g., loan of gait aids or positioning devices) from KidsAbility was also identified as a solution to enhance experience. Currently, families accessing services virtually need to come on site to pick up these physical materials, which one caregiver said, “defeated the purpose of online” (Caregiver P5-4).

## 4. Discussion

**Figure 1:**
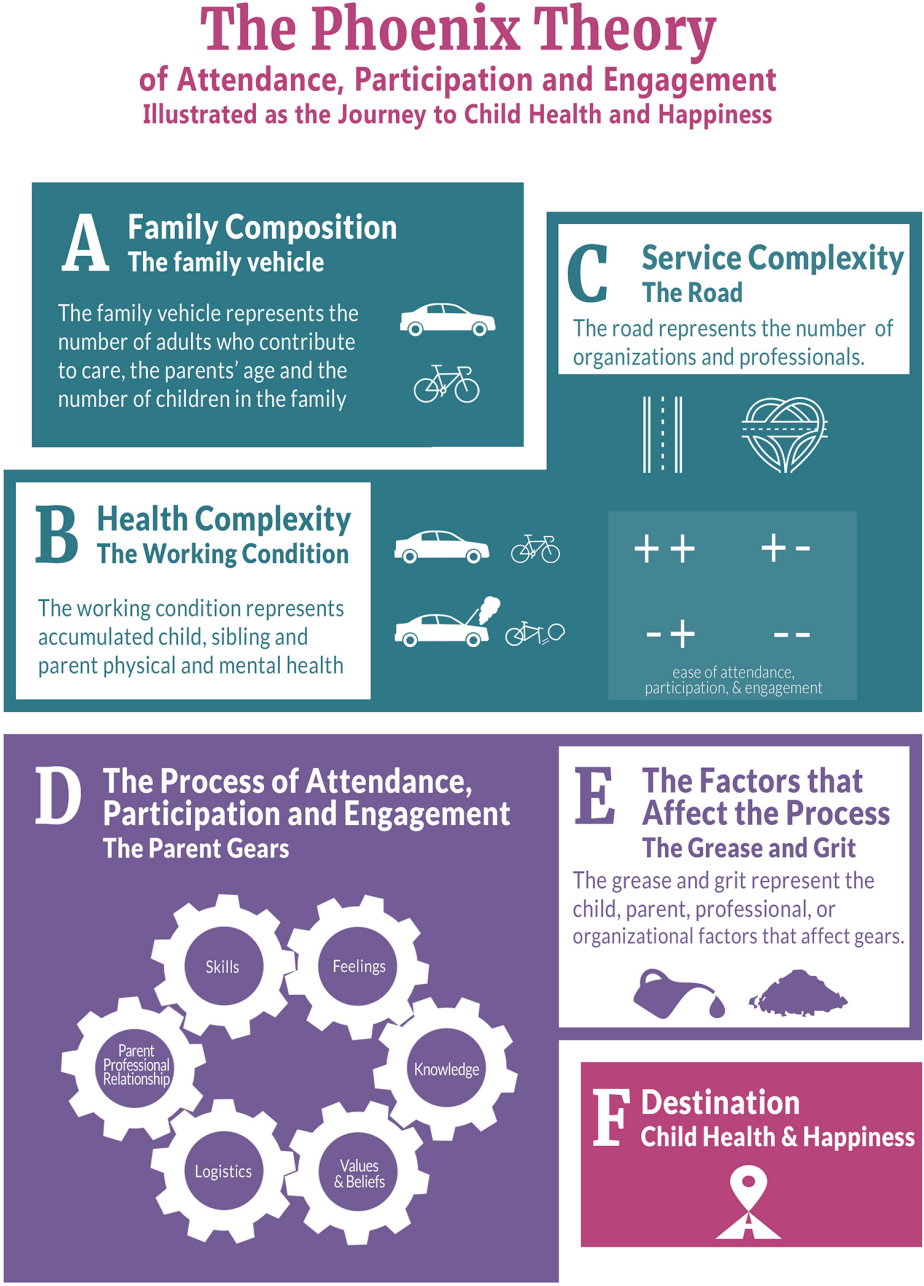
The Phoenix theory of attendance, participation and engagement (15). © 2019 The Author(s). Published by Informa UK Limited, trading as Taylor & Francis Group. Reproduced with permission from Informa UK Limited through PLSclear.

The aim of this project was to determine what solutions could be co-designed to enhance pediatric telerehabilitation experiences by understanding and incorporating the experiences of caregivers, clinicians and management. The 3C's emerged from the codesign process as key factors that influence engagement in telerehabilitation before, during and after a visit. The co-designed solutions were proposed to improve families access and engagement in telerehabilitation services. The Phoenix Theory of Attendance, Participation and Engagement (the Phoenix Theory) depicted in Figure 1, examined missed appointments in the context of in-person pediatric rehabilitation at KidsAbility and provided a theoretical foundation our work (15). The Phoenix Theory describes six interconnected gears that influence the process of parents attending, participating and engaging in therapy including: skills, feelings, knowledge, values and beliefs, logistics, and the parent-professional relationship (15). Additionally, the theory describes factors at the level of the child, parent, professional or organization that interact with the parent gears as either grit (inhibits gear movement) or grease (facilitates gear movement) (15). Although not developed or tested in the context of pediatric telerehabilitation, we see alignment between our findings and some the constructs of this theory. The trustworthiness of our findings, including the co-designed solutions, is enhanced through theoretical triangulation with components of the Phoenix Theory.

The connection between the clinician and the family was identified in our findings as a factor impacting experience with telerehabilitation. A desire to establish and maintain this connection is evident in the co-designed solutions developed (i.e., conversation with caregivers about their child). The element of connection discussed in our findings is akin to the parent-professional gear represented in the Pheonix Theory (15). According to the Phoenix Theory, a trust-based relationship and connection between clinician and caregiver enhances agreement between these two parties related to how to move forward in therapy (15). Relationships and collaborations have been recognized as indicators of levels of engagement in therapy (17). In a study exploring engagement and therapeutic alliance in pediatric telerehabilitation, rapport, connection and collaboration were identified as influencing caregiver engagement in telerehabilitation services (10). These findings are further supported by a qualitative systematic review exploring engagement in early intervention telerehabilitation, where building rapport between caregiver and clinician was linked to improved therapeutic outcomes, facilitating open communication and enhancing caregiver buy-in (21). This review highlighted the benefit of establishing early therapeutic rapport, suggesting relationship building should begin prior to telerehabilitation commencing (21), aligning with the co-designed solution recommending an appointment between clinician and caregiver prior to starting teletherapy with the child.

The Phoenix Theory identifies resources as one of the factors that can add grit or grease, influencing how the parent gears operate (15). Resources as described by the Phoenix Theory, include information and organizational supports, amongst other resource groupings (15). Co-designed solutions geared toward developing video tutorials and text-based resources about telerehabilitation services align closely with the Phoenix Theory's informational resources, which are factors that can influence engagement and experience with services. Examples of resources related to organizational supports are the possible adaptations and flexibility of service options (15). Our findings indicate the need for clinicians to consistently communicate service options available to families and a desire from caregivers to have a choice in their preferred service model. In a 2023 realist evaluation of telehealth in children with neurodisabilities, the importance of offering caregivers the choice to participate in telerehabilitation as part of a hybrid model (i.e., option for in person appointments, telerehabilitation appointments or both) was critical to their acceptance of telerehabilitation as a meaning option for service (40).

Communication and expectations were closely linked concepts in our findings and are represented individually as factors influencing the parent gears in the Phoenix Theory (15). Many of the co-designed solutions from our project aimed to establish consistency in the content and quality of the communication between the organization and families, with the hopes of aligning expectations for telerehabilitation service. The co-designed solutions targeted process change and resource development to achieve improvements in communication. The Phoenix Theory describes higher levels of parent engagement in services when there is alignment between what they expected the service to be like and what they received (15). Expectations are closely connected to the knowledge parent gear in the Phoenix Theory (15). In our findings, parents expressed not knowing what to expect with regards to telerehabilitation services, sharing that this knowledge was not adequate or consistently communicated and experiences with one clinician could be very different from service with a different clinician. Literature on caregiver expectations of therapy shows that caregivers enter into therapeutic interactions with expectations for their child, the clinician, the service organization and themselves (41). An ethnographic study exploring engagement in outpatient pediatric rehabilitation reported that engagement in therapy increases when expectations for therapy are aligned between caregiver and clinician, specifically when there are clear expectations about roles within the sessions (17).

Communication has been identified as one of the most important factors influencing parent engagement (15) and according to our findings is highly influential to the telerehabilitation experience. Collaborative, two-way communication, where caregivers feel listened to and feel their input is valued has been identified as critical to engagement in pediatric telerehabilitation services (10, 21, 40). With the recognition that it will take more of the clinicians' time, the use of multimodal communication approaches within and outside of telerehabilitation appointments has been identified as instrumental in facilitating engagement and connection (21). The need for using a multimodal approach to communication (e.g., using a combination of email and phone communication according to preference), tailored to each families' context is recognized in our findings and the co-designed solutions.

A limitation of this work is that the sample can only be described from a relatively small set of demographic questions focused on maximizing the diversity of the sample according to the KidsAbility context (e.g., KidsAbility site, clinical discipline, age of child receiving service, access to reliable internet connection). Additional demographic information such as income level or parent education level, was not collected and therefore potentially limits the transferability to other contexts. A strength of this work is that it included a broad range of perspectives including caregivers, clinicians, KidsAbility management and interdisciplinary researchers in all phases of the project. Due to time and resource constraint, there was not opportunity to review the co-designed solutions with participants who took part in the co-design groups, however they were validated with the steering committee members, some of which have lived and living experience with telerehabilitation services. We acknowledge that although the project is grounded in the field of pediatric rehabilitation, the child and youth voice is not represented in our work and should be incorporated into future research in this area. A possible avenue for gaining insight into youth experience with telerehabilitation is engaging with the established KidsAbility Youth Advisory Council for future projects. Although the project was completed with a single site potentially limiting the transferability of the findings, this allowed for a rich understanding of the study context and the development of solutions relevant to KidsAbility.

To date, our project has developed co-designed solutions aiming to enhance experiences with pediatric telerehabilitation. The relevance and validity of these solutions to practice has been explored through examining their relationships to theory and current evidence. Next steps of this project are to work alongside KidsAbility to implement and evaluate the impact of these solutions on organizational practices and user experience with telerehabilitation services in this setting. Evidence-based knowledge products developed to support pediatric telerehabilitation appointments, such as the Telerehabilitation Hub for Children with Disabilities and their Families (42) will be explored to operationalize the solutions developed from our co-design work in the KidsAbility context. Additionally, our team has plans for disseminating information about the co-designed solutions across an established pan-Canadian network of research and clinical pediatric rehabilitation organizations.

## Data Availability

The datasets presented in this article are not readily available. The data for this study cannot be shared to protect the privacy and confidentiality of participants. The dataset is unavailable to be requested.

## References

[1] Miller AR Mâsse LC Shen J Schiariti V Roxborough L. Diagnostic status, functional status and complexity among Canadian children with neurodevelopmental disorders and disabilities: a population-based study. Disabil Rehabil. (2013) 35:468–78. 10.3109/09638288.2012.699580 22794277

[2] Charters T Schimmele C Arim R. A profile of children with affirmative responses to the 2016 census questions on difficulties with activities of daily living. Stat Canada. (2022) 2:1–11. 10.25318/36280001202200300006-eng

[3] Accardo J Shapiro BK. Neurodevelopmental disabilities: beyond the diagnosis. Semin Pediatr Neurol. (2006) 12:242–9. 10.1016/j.spen.2005.12.006 16780295

[4] Anaby DR Law M Feldman D Majnemer A Avery L. The effectiveness of the pathways and resources for engagement and participation (PREP) intervention: improving participation of adolescents with physical disabilities. Dev Med Child Neurol. (2018) 60:513–9. 10.1111/dmcn.13682 29405282

[5] Chen CC Heinemann AW Bode RK Granger CV Mallinson T. Impact of pediatric rehabilitation services on children’s functional outcomes. Am J Occup Ther. (2004) 58:44–53. 10.5014/ajot.58.1.44 14763635

[6] Empowered Kids Ontario. Access to Service. Child Rehab by Numbers (2016). Available at: https://empoweredkidsontario.ca/en/BtNAccesstoService (Accessed December 6, 2020).

[7] Camden C Silva M. Pediatric teleheath: opportunities created by the COVID-19 and suggestions to sustain its use to support families of children with disabilities. Phys Occup Ther Pediatr. (2021) 41:1–17. 10.1080/01942638.2020.1825032 33023352

[8] Rosenbaum PL Silva M Camden C. Let’s not go back to ‘normal’! lessons from COVID-19 for professionals working in childhood disability. Disabil Rehabil. (2021) 43:1022–8. 10.1080/09638288.2020.1862925 33355010

[9] Parmanto B Saptono A. Telerehabilitation: state-of-the-art from an informatics perspective. Int J Telerehabilitation. (2009) 1:73–84. 10.5195/ijt.2009.6015 PMC4296781 25945164

[10] Fairweather GC Lincoln M Ramsden R Bulkeley K. Parent engagement and therapeutic alliance in allied health teletherapy programs. Health Soc Care Community. (2021) 00:1–10. 10.1111/hsc.13235 33586838

[11] Ogourtsova T Boychuck Z O’Donnell M Ahmed S Osman G Majnemer A. Telerehabilitation for children and youth with developmental disabilities and their families: a systematic review. Phys Occup Ther Pediatr. (2023) 43:129–75. 10.1080/01942638.2022.2106468 36042567

[12] Caprì T Nucita A Iannizzotto G Stasolla F Romano A Semino M Telerehabilitation for improving adaptive skills of children and young adults with multiple disabilities: a systematic review. Rev J Autism Dev Disord. (2021) 8:244–52. 10.1007/s40489-020-00214-x

[13] Lindsay S Ragunathan S Kingsnorth S Zhou C Kakonge L Cermak C Understanding the benefits and challenges of outpatient virtual care during the COVID-19 pandemic in a Canadian pediatric rehabilitation hospital. Disabil Rehabil. (2023):1–9. 10.1080/09638288.2023.2221902 37306595

[14] King G Chiarello LA McLarnon MJW Ziviani J Pinto M Virginia Wright F A measure of parent engagement: plan appropriateness, partnering, and positive outcome expectancy in pediatric rehabilitation sessions. Disabil Rehabil. (2021) 44:3459–68. 10.1080/09638288.2020.1864036 33390023

[15] Phoenix M Jack SM Rosenbaum PL Missiuna C. A grounded theory of parents’ attendance, participation and engagement in children’s developmental rehabilitation services: part 2. The journey to child health and happiness. Disabil Rehabil. (2020) 42:1251–60. 10.1080/09638288.2018.1555618 30669898

[16] Phoenix M Jack SM Rosenbaum PL Missiuna C. Parents’ attendance, participation and engagement in children’s developmental rehabilitation services: part 1. Contextualizing the journey to child health and happiness. Disabil Rehabil. (2020) 42:2141–50. 10.1080/09638288.2018.1555617 30669893

[17] King G Chiarello LA Ideishi R Ziviani J Phoenix M McLarnon MJW The complexities and synergies of engagement: an ethnographic study of engagement in outpatient pediatric rehabilitation sessions. Disabil Rehabil. (2019) 43:2353–65. 10.1080/09638288.2019.1700562 31847621

[18] Camden C Pratte G Fallon F Couture E Berbari J Tousignant M. Diversity of practices in telerehabilitation for children with disabilities and effective intervention characteristics: results from a systematic review. Disabil Rehabil. (2020) 42:3424–36. 10.1080/09638288.2019.1595750 30978110

[19] Alonazi A. Effectiveness and acceptability of telerehabilitation in physical therapy during COVID-19 in children: findings of a systematic review. Children. (2021) 8:1101. 10.3390/children8121101 34943295 PMC8700182

[20] Dostie R Gaboury I Cinar E Camden C. Acceptability of pediatric telerehabilitation interventions provided by physical therapists and occupational therapists-A scoping review. Phys Occup Ther Pediatr. (2022) 42:615–34. 10.1080/01942638.2022.2064203 35440285

[21] Retamal-Walter F Waite M Scarinci N. Exploring engagement in telepractice early intervention for young children with developmental disability and their families: a qualitative systematic review. Disabil Rehabil Assist Technol. (2022) 18:1–14. 10.1080/17483107.2022.2048098 35287526

[22] Ballantyne M Rosenbaum PL. Missed appointments: more complicated than we think. Paediatr Child Health (Oxford). (2017) 22:164–5. 10.1093/pch/pxx039 PMC5804886 29479206

[23] Phoenix M. Chapter 17 Service provision for hard-to-reach families: what are our responsibilities? In: Rosenbaum PL Ronen GM Racine E Johannesen J Dan B, editors. Ethics in child health. United Kingdom: Mac Keith Press (2016). p. 193–201.

[24] Mulvale G Moll S Miatello A Murray-Leung L Rogerson K Sassi RB. Co-designing services for youth with mental health issues: novel elicitation approaches. Internaltion J Qual Methods. (2019) 18:1–13. 10.1177/1609406918816244

[25] Moll S Wyndham-West M Mulvale G Park S Buettgen A Phoenix M Are you really doing a “codesign”? critical reflections when working with vulnerable populations. BMJ Open. (2020) 10:1–5. 10.1136/bmjopen-2020-038339 PMC7640510 33148733

[26] Bate P Robert G. Experience-based design: from redesigning the system around the patient to co-designing services with the patient. Qual Saf Heal Care. (2006) 15:307–10. 10.1136/qshc.2005.016527 PMC2565809 17074863

[27] Bate P Robert G. Bringing user experience to healthcare improvement: The concepts, methods and practices of experience-based design. Oxford: Radcliffe Publishing (2007).

[28] Donetto S Pierri P Tsianakas V Robert G. Experience based co-design and healthcare improvement: realizing participatory design in the public sector. Des J. (2015) 18:227–48. 10.2752/175630615X14212498964312

[29] Mulvale G Moll S Miatello A Robert G Larkin M Palmer VJ Codesigning health and other public services with vulnerable and disadvantaged populations: insights from an international collaboration. Heal Expect. (2019) 22:284–97. 10.1111/hex.12864 PMC6543156 30604580

[30] Tang CY Turczyniak M Sayner A Haines K Butzkueven S O’connell HE. Adopting a collaborative approach in developing a prehabilitation program for patients with prostate cancer utilising experience-based co-design methodology. Support Care Cancer. (2020) 28:5195–202. 10.1007/s00520-020-05341-z/Published 32072326

[31] Zoom. Zoom Video Communications I (2023).

[32] Google. Google Jamboard. Available at: jamboard.google.com

[33] Harris PA Taylor R Minor BL Elliott V Fernandez M O’Neal L The REDCap consortium: building an international community of software platform partners. J Biomed Inform. (2019) 95:103208. 10.1016/j.jbi.2019.103208 31078660 PMC7254481

[34] Mulvale G Miatello A Green J Tran M Roussakis C Mulvale A. A COMPASS for navigating relationships in co-production processes involving vulnerable populations. Int J Public Adm. (2021) 44:790–802. 10.1080/01900692.2021.1903500

[35] Holskey MP Rivera RR. Optimizing nurse engagement: using liberating structures for nursing professional practice model development. J Nurs Adm. (2020) 50:468–73. 10.1097/NNA.0000000000000918 32826516

[36] Faughnan M Murphy L. Liberating structures for pluriversal world-making. Dismantling/Reassembling Pivot 2021 (Virtual Conference). p. 1–21. 10.21606/pluriversal.2021.0015

[37] Mallette C Rykert L. Promoting positive culture change in nursing faculties: getting to maybe through liberating structures. J Prof Nurs. (2018) 34:161–6. 10.1016/j.profnurs.2017.08.001 29929794

[38] Elo S Kyngäs H. The qualitative content analysis process. J Adv Nurs. (2008) 62:107–15. 10.1111/j.1365-2648.2007.04569.x 18352969

[39] QSR International Pty Ltd. NVivo (2022).

[40] Graham F Williman J Sutherland L Wijninckx M. Telehealth delivery of paediatric rehabilitation for children with neurodisability: a mixed methods realist evaluation of contexts, mechanisms and outcomes. Child Care Health Dev. (2023) 49:156–69. 10.1111/cch.13028 35778916 PMC10084377

[41] Phoenix M Smart E King G. “I didn’t know what to expect”: describing parents’ expectations in children’s rehabilitation services. Phys Occup Ther Pediatr. (2020) 40:311–29. 10.1080/01942638.2019.1665155 31530201

[42] Ogourtsova T. TelereHUB-CHILD: an online integrated knowledge translation tool to optimize telerehabilitation evidence-based practices for children with disabilities and their families. Front Rehabil Sci. (2023) 4. 10.3389/fresc.2023.1139432 PMC10083307 37050918

